# Coagulopathy associated with cell salvage transfusion following cerebrovascular surgery

**DOI:** 10.12669/pjms.296.3750

**Published:** 2013

**Authors:** Jianqiao Zheng, Li Du, Guizhi Du, Bin Liu

**Affiliations:** 1 Jianqiao Zheng, MD, Department of Anesthesiology, West China Hospital of Sichuan University, No. 37, Guo Xue Alley, Chengdu 610041, Sichuan, China.; 2 Li Du, MD, Department of Anesthesiology, Sichuan Cancer Hospital, No.55, People's South Road, Chengdu 610041, Sichuan, China.; 3 Guizhi Du, MD, Department of Anesthesiology, West China Hospital of Sichuan University, No. 37, Guo Xue Alley, Chengdu 610041, Sichuan, China.; 4 Bin Liu, MD, PhD, Department of Anesthesiology, West China Hospital of Sichuan University, No. 37, Guo Xue Alley, Chengdu 610041, Sichuan, China.

**Keywords:** Activated coagulation time, Cell saver, Coagulopathy, Heparin

## Abstract

A 35-year-old man was scheduled for dural arteriovenous fistula resection for vascular malformation under general anesthesia and a cell saver device was employed. The patient suffered from massive bleeding for the rupture of arteriovenous malformations from the beginning of the operation and 1000 mL cell-saved blood was transfused. After autologous blood transfusion and fluid resuscitation, blood oozed significantly from the surgical wounds, and the administration of cryoprecipitate and fibrinogen has no effect. The value of the activated coagulation time (ACT) increased to 999s. Considering the residual heparin in the autologous blood, ninety mg of protamine was intravenously injected, then 5 minutes later the ACT dropped to 147s. After the therapy, the surgical procedure was performed smoothly. The activated partial thromboplastin time (APTT) and the thrombin time (TT) of the postoperative venous blood was 18.9 s and 53.6 s respectively. Two days later, the APTT and the TT decreased to 12.1 s and 32.7 s without special treatment. The patient was discharged home without complications and well follow-up.

## INTRODUCTION

Cell salvage in the surgery is considered beneficial in reducing allogeneic blood transfusion.^1^ Multiple randomised controlled trials and meta-analyses have indicated a significant decrease in allogenic transfusion with the use of cell salvage techniques.^2^ In most circumstances, cell salvage is safe for the coagulation function and the effect of residual heparin from the cell saver would be negligible.^3^ The platelets and coagulation factors are depleted during the washing of red cells by the cell saver. The processing of spilled blood during the cell salvage may result in depletion coagulopathy in patients with massive bleeding. In this condition, washing of red cells by the cell saver and retransfusion of suspended red cells can occasionally cause coagulopathy, particularly when using high concentration heparin saline to wash the spilled blood.^4^

Consequently, avoidance of cell salvage transfusion-associated coagulopathy is critical for the patients with massive bleeding. We report this case about coagulopathy associated with cell salvage transfusion and wish to highlight the importance of heparin-induced bleeding that can be overlooked in these situations. Consent was obtained from the patient prior to submission of this case for publication.

## CASE REPORT

A 35-year-old, American Society of Anesthesiologists Class III, 65-kg man suffering from headache, hypomnesia and unresponsiveness for 5 months, was scheduled for dural arteriovenous fistula resection under general anesthesia for the right temporal lobe hemorrhage caused by vascular malformation and dural arteriovenous fistula. His preoperative physical examinations were unremarkable. Besides the routine monitors, a right radial arterial line was inserted after the Allen test negative for the invasive blood pressure monitoring. The cell saver was routinely prepared preoperatively to provide salvage capability in the event of a major hemorrhage at our institution. After anesthesia induction and intubation, the surgical procedure was started. When dissecting the bone flap during the craniotomy, the blood loss exceeded 600 mL in a short time.

The surgeon applied the tamponade hemostasis, and simultaneously we took the rapid fluid replacement, prepared blood products, monitored the artery blood gas and injected metaraminol intravenously according the invasive blood pressure. One hour later, when the hemodynamics stabilized after the treatment, the operation was continued. But after removing the bone flaps, massive bleeding occurred once again. The compression hemostasis was applied. We administrated the patient both allogenic and autologus blood transfusion as well as fluid resuscitation. In addition, tranexamic acid and hemocoagulase were delivered intravenously. Also metaraminol and noradrenaline were applied. In total, 4 units of packed red cells, 400 mL of fresh frozen plasma, 1000 mL of cell salvage, 3100 mL of crystalloid solution and 2500 mL of colloid solution were administrated. When the hemodynamic parameters were stable after the treatments, the oozing of blood in surgical wounds occurred. Then 10 units of cryoprecipitate and 1 g of fibrinogen were administrated, but it had no effect. Subsequently we monitored the activated coagulation time (ACT) of the venous blood, and the value was 999s.

Considering the possibility of the residual heparin in the cell salvage, 90 mg of protamine was injected intravenously, and 5 minutes later the ACT dropped to 147 s. After the therapy, the surgical procedure was performed smoothly. The operation took 6 hrs, 4 units of packed red cells, 400 mL of fresh frozen plasma, 1700 mL of cell salvage, 4100 mL of crystalloid solution and 3000 mL of colloid solution were transfused totally, and the urine was 1000 mL. After the procedure was over, the patient went back to the intensive care unit (ICU) with an activated partial thromboplastin time (APTT) of 18.9 s and a thrombin time (TT) 53.6 s. Two days later, APTT and TT decreased to 12.1 s and 32.7 s respectively without special treatment. The patient was discharged home after 13 days without complications and well on follow-up.

## DISCUSSION

Cell saver blood is a useful adjunct to reduce the perioperative allogenic blood transfusion. As the standard protocol practiced at our institution, the heparinised saline containing 12,500 IU of heparin in 1 liter of 0.9% saline at a rate of 100 mL per hour is used to prevent thrombogenesis during the collection of blood from the operating field. The washing program used in the cell saver involved a 5:1 ratio between the saline wash and blood. As the study confirms, after processing the spilled blood by the system, the red cell suspension that is transfused back to the patient may contain about 0.002% of the pre-wash heparin.^3^ Therefore, the cell saver can be used safely idn patients without concerning the residual heparin when properly processed. If hemorrhea takes place and repetitive washing is absolutely necessary, the loss of plasma proteins, platelets, and coagulation factors occur with an automatic blood recovery system. Previous studies revealed that if the amount of autologous transfusion is less than 3000 mL, coagulation remains normal.^5^ The practicing of cell saver depletes the platelets and coagulation factors. In addition, the fluid therapy and massive transfusion during massive bleeding will cause the coagulation dysfunction by hemodilution. Repeated return of washed red cells and residual heparin from the cell saver can worsen the coagulopathy. Thus, timely reversal of heparin in such cases may reduce further excessive blood loss.

The dose of heparin in the autologous blood will vary dependent on transfusion volume, concentration and drip rate of anticoagulant heparin solution. But we found the estimation is very cumbersome in clinical practice. Heparin anticoagulation is commonly monitored with ACT. The ACT may be prolonged by hemodilution, but it still provides an extremely useful, fairly reliable bedside method for monitoring heparin therapy and the adequacy of anticoagulation.^6^ Therefore, clinicians would have to use protamine to reversal the residual heparin according to the ACT values in this situation. In order to administer the precise dosage of protamine to revasal the residual heparin, it is also necessary to monitor the preoperative ACT value. In addition, a modified thrombin clotting time test has been used as a cheap and reliable marker for heparin contamination in obstetric intraoperative cell salvage.^7^ A recent study revealed that the Sonoclot analyzer is a reliable and sensitive device for assessment of the heparinization levels within 20 min.^8^ Theses methods can be also used for monitoring the residual heparinization levels after the autologous blood transfusion.

The appropriate concentration of the anticoagulant heparin solution and the degree of washing performed by the cell saver are vital to preventing the residual heparin from the autologous blood. The concentration of the anticoagulant heparin solution was 50,000 IU/L, even 30,000 IU/L in some report.^4^ But, in our hospital, 25,000 IU/L of the anticoagulant heparin solution is safe and effective during the cell salvage. A 5:1 volume ratio between the saline wash and blood is preferred for the washing program.

Sodium citrate 3% has been used as an alternative to heparin as anticoagulation in cell salvage.^9^ Also, sodium citrate 4% was as effective as heparin in the maintenance of catheter patency for long-term hemodialysis.^10^ In addition, it was associated with fewer catheter-related Infections, bleeding risk and lower increase of the APTT when compared with heparin.^10^ In our hospital, heparin was the most commonly used anticoagulant in the autotransfusion in patients undergoing orthopaedic spinal operations, but the surgeons always complained that autotransfusion was associated with a more significant derangement of the postoperative drainage output than homologous blood. Then we tried the sodium citrate 4% as an alternative to heparinised saline, and fortunately the drainage output declined after the alternation. And no perioperative complications were observed.

## CONCLUSION

As the increasing popularity of the cell saver, more attention should be focused on the patients’ coagulation function after the autologous blood transfusion, especially in the patients with massive bleeding. We recommended that the monitoring of the residual heparin by the ACT value, modified thrombin clotting time test or the Sonoclot analyzer before and after the autologous blood transfusion is an absolutely necessary procedure. Furthermore, sodium citrate 4% could be used as an alternative to heparin in cell salvage. More importantly, it is necessary to develop the clinical practice guidelines on the cell salvage as soon as possible.

## Notes

***Funding:*** None declared

## References

[1] Aning J, Dunn J, Daugherty M, Mason R, Pocock R, Ridler B (2012). Towards bloodless cystectomy: a 10-year experience of intra-operative cell salvage during radical cystectomy. BJU Int.

[2] Carless PA, Henry DA, Moxey AJ, O'Connell D, Brown T, Fergusson DA (2012). Cell salvage for minimising perioperative allogeneic blood transfusion. Cochrane Database Syst Rev.

[3] Sistino JJ, Owitz D, Mongero LB (1992). Heparin washout in the pediatric cell saver bowl. J Extra Corpor Technol.

[4] Rollins KE, Trim NL, Luddington RJ, Colah S, Klein A, Besser MW (2011). Coagulopathy associated with massive cell salvage transfusion following aortic surgery. Perfusion.

[5] Jagoditsch M, Pozgainer P, Klingler A, Tschmelitsch J (2006). Impact of blood transfusions on recurrence and survival after rectal cancer surgery. Dis Colon Rectum.

[6] Marmur JD, Anand SX, Bagga RS, Fareed J, Pan CM, Sharma SK (2003). The activated clotting time can be used to monitor the low molecular weight heparin dalteparin after intravenous administration. J Am Coll Cardiol.

[7] Sullivan IJ, Hicks MK, Faulds JN, Carson PJ, Noble RS (2012). A modified thrombin clotting time test as a quality control marker for heparin contamination in obstetric intraoperative cell salvage. Transfusion Med.

[8] Lee B, Al-Waili N, Butler G, Salom K (2011). Assessment of heparin anticoagulation by Sonoclot Analyzer in arterial reconstruction surgery. Technology Health Care.

[9] Moltermans Y, Vermaut G, Van Aken H, Goossens W, Boogaerts M (1994). Quality of washed salvaged red blood cells during total hip replacement: a comparison between the use of heparin and citrate as anticoagulants. Anesth Analg.

[10] Yon CK, Low CL (2013). Sodium citrate 4% versus heparin as a lock solution in hemodialysis patients with central venous catheters. Am J Health Syst Pharm.

